# Simulation environment and graphical visualization environment: a COPD use-case

**DOI:** 10.1186/1479-5876-12-S2-S7

**Published:** 2014-11-28

**Authors:** Mercedes Huertas-Migueláñez, Daniel Mora, Isaac Cano, Dieter Maier, David Gomez-Cabrero, Magí Lluch-Ariet, Felip Miralles

**Affiliations:** 1 Barcelona Digital Technology Centre, 08018 Barcelona, Spain; 2 Hospital Clinic, IDIBAPS, Universitat de Barcelona, 08036 Barcelona, Spain; 3 Biomax Informatics, AG, D-82152 Planegg, Germany; 4 Unit of Computational Medicine, Department of Medicine, Karolinska Institutet, 171 77 Solna, Sweden

**Keywords:** Simulation environment, deterministic model, probabilistic model, interoperability, knowledge base, data base, case

## Abstract

**Background:**

Today, many different tools are developed to execute and visualize physiological models that represent the human physiology. Most of these tools run models written in very specific programming languages which in turn simplify the communication among models. Nevertheless, not all of these tools are able to run models written in different programming languages. In addition, interoperability between such models remains an unresolved issue.

**Results:**

In this paper we present a simulation environment that allows, first, the execution of models developed in different programming languages and second the communication of parameters to interconnect these models. This simulation environment, developed within the Synergy-COPD project, aims at helping and supporting bio-researchers and medical students understand the internal mechanisms of the human body through the use of physiological models. This tool is composed of a graphical visualization environment, which is a web interface through which the user can interact with the models, and a simulation workflow management system composed of a control module and a data warehouse manager. The control module monitors the correct functioning of the whole system. The data warehouse manager is responsible for managing the stored information and supporting its flow among the different modules.

This simulation environment has been validated with the integration of three models: two deterministic, i.e. based on linear and differential equations, and one probabilistic, i.e., based on probability theory. These models have been selected based on the disease under study in this project, i.e., chronic obstructive pulmonary disease.

**Conclusion:**

It has been proved that the simulation environment presented here allows the user to research and study the internal mechanisms of the human physiology by the use of models via a graphical visualization environment. A new tool for bio-researchers is ready for deployment in various use cases scenarios.

## Introduction

In the framework of systems medicine, data comes from different biological or medical levels [1]. The aim of researchers is to understand the causes of different processes in the human body, with the support of mathematical and computational models.

A *model *may be defined as a simplified representation of the real world. Models describe systems employing parameter description and parameter interaction [1]. In this work, we have considered two modelling paradigms: a deterministic one, based on linear and ordinary differential equations, and a probabilistic one, based on probability theory.

This SE has been developed within the Synergy-COPD project under the 7th Framework Program [2-4]. The objectives of the project are to integrate five existing computer models and develop a *simulation environment *(SE) which will allow and facilitate the development, implementation and deployment of systems medicine. A *graphical visualisation environment *(GVE) will allow users to access the *knowledge base *(KB) where all the information about the models is stored, interact with them and carry out simulations in a more intuitive way with respect to the state of the art.

After a revision of the state of the art in the area of simulation tools for physiological models (see Section state of the art), considering the kind of knowledge [5] and the models included in the project [1,6-8], we developed this SE. The SE is able to run models written in different programming languages and communicate parameters among models.

The paper is organized as follows. The state of the art section contains a review of existing tools considering their capability to run physiology models. The architecture of the simulation environment section describes the structure and functionalities of the two main modules that compose the SE. The interoperability section describes how the interoperability among modules is reached. The workflow section describes the sequence of actions to be carried out to run a simulation. The validation section describes how this SE was validated by the use of physiological models related to COPD, and the conclusions and future work section adds concluding remarks and the direction of future work.

## State of the art

Mathematical models and simulations are widely used in the study of physiological processes. Presently several tools can be found in the literature with this aim. In this section, the most relevant of these are reviewed. Table 1 shows the evaluation of these tools based on three aspects: (1) parameter communication, (2) model description and model execution result storage and (3) execution of models in different programming languages.

**Table 1:** Comparision of existing simulation tools.

Simulation tool	Parameter communication	Data storage	Integration of models in different compiled programming languages
**JSim**	-	-	+
**PhysioDesigner**	-	-	-
**Cytosolve**	+	+	-
**Chaste**	-	-	-
**HumMod**	-	+	-
**MoBi**	+	+	-

This table compares the tools described in the section State of the Art in terms of communication of paramenters, data storgae and integration of models in different programming languages.

• JSim [9] is a tool developed within the National Simulation Resource Physiome project to run models written mainly in *mathematical modelling language *(MML), *systems biology markup language *(SBML) and *CellML markup language*. The connection of models written in Java or C++ requires the use of a specific library developed in MML. JSim has its own data base to store models but there is no support of parameter communication between the models.

• PhysioDesigner [10] is a platform developed by the University of Osaka. Models are developed in *physiological markup language *(PHML). *Flint *is the simulation tool developed within PhysioDesigner to run models written in PHML/ISML, CellML and SBML. This simulation environment does not run models in other programming languages than PHML/ISML, CellML and SBML.

• Cytosolve [11] is a system that integrates dynamically the computations of smaller models that can be run in different machines. Models are mainly written in SBML and CellML. The web services parse and detect naming conflicts and common reaction pathways among models. This information is stored in the ontology of the data base. Once the user has specified the models and the conflicts are solved, the integration of the models can start. Cytosolve connects computational results after model execution using a messaging passing system based on XML across the collaborative models. This tool runs models mainly written in SMBL and CellML.

• The Cancer, Heart and Soft Tissue Environment (Chaste) [12] supports the simulation of spatial models. It allows the visualization and post-processing of simulation results. Chaste is only supported in Linux/Unix platforms. Chaste supports models developed in C++. It does not store data and there is no support of parameter communication between the models.

• HumMod [13] is a simulation and modelling tool. Models description is represented in XML. HumMod is composed of two modules: model documentation, which contains the XML files that correspond to models description, and model navigator, which displays the relationships between variables. The knowledge base contains 5000 variables obtained from peer reviewed physiological papers. HumMod does not support models developed in other programming languages.

• MoBi [14] is a tool to model biological processes and drug actions. MoBi works by using PK-Sim another package for *physiologically-based pharmacokinetic *(PBPK) modelling. This tool allows building and simulating physiology models, export and import to other modelling languages and visualization of results. MoBi can be integrated into MATLAB and R for sensitivity and complex analysis. Models can only be defined in a specific XML format.

As presented in Table 1 some of the tools reviewed here do not support some of the evaluation aspects stated in the beginning of the section. The contributions of our SE to the SoA are based on its capability to run models programmed in different compiled programming languages allowing the exchange of parameters values between models. This SE is able to request and retrieve information from a complex *knowledge base *(KB), where all the information about the models is stored. The SE processes that information to present it to the user via a web-based interface which allows him to carry out simulations in a more intuitive way with respect to the state of the art. Our SE has also a data base to register and keep track on user's activity while logged in.

## Architecture of the simulation environment

Considering the complexity of the models included in the Synergy-COPD project, the complexity of the knowledge involved and the necessity to communicate parameters among models, the *simulation environment *was developed to satisfy these requirements.

**Figure 1:**
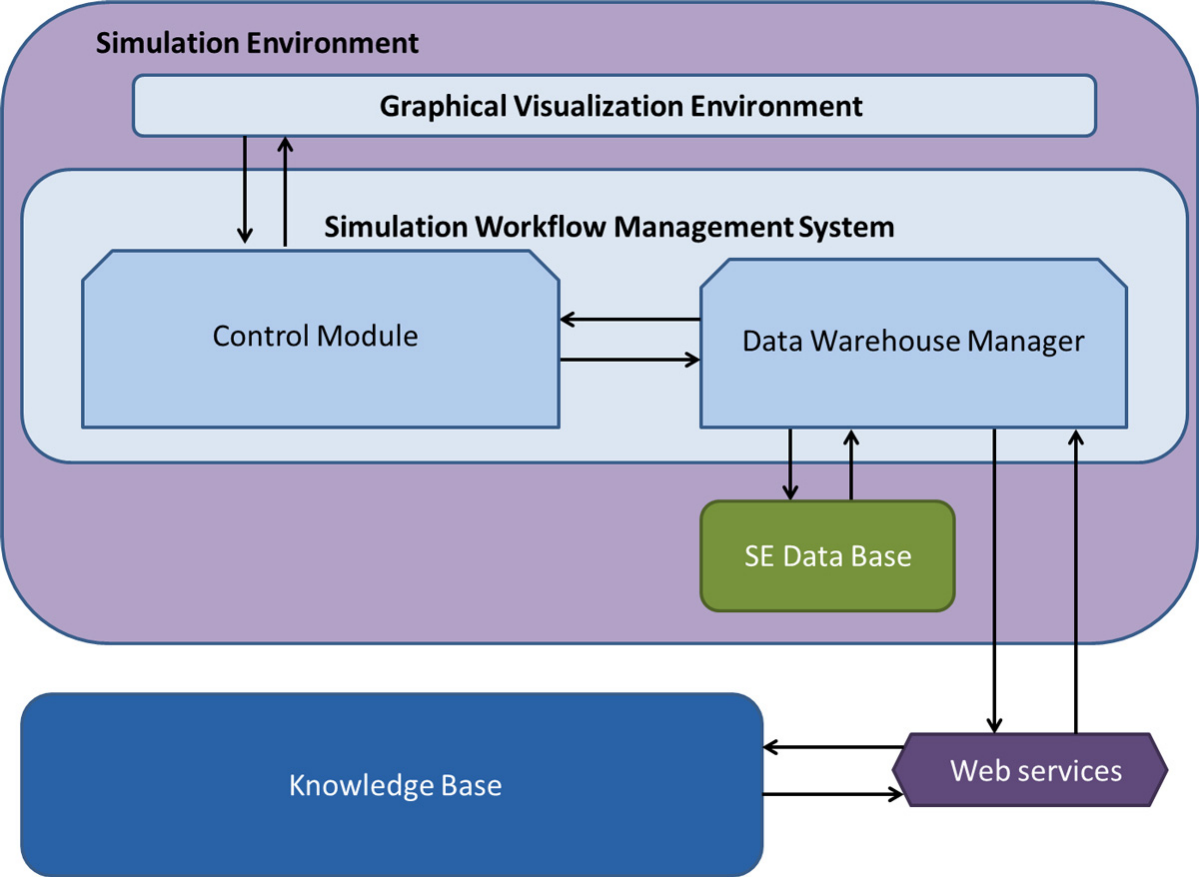
**Architecture of the SE**.

The SE described in this paper is composed of two main modules as presented in Figure 1: the *simulation workflow management system *(SWoMS) and the *graphical visualization environment *(GVE). The GVE is the visual part of the system, through which the user can interact with the models. The SWoMS is composed of two modules: the *data warehouse manager *and the *control module*.

The SE interacts with an external module, the (KB) [15], which stores all the information related to models and *cases*. A *case *in the SE is defined as the necessary information to accurately describe the (real or hypothetical) status of a human being. This information also corresponds to a set of variables included in the models. *Cases *can correspond to patients or can correspond to the result of simulations in which the values of certain variables have been modified. Variables can be represented differently in different models and the KB includes the mappings among these different representations, which include the conversion of units. While details of the mapping are described elsewhere [5], we will briefly summarise the KB status to facilitate the understanding of the following sections. The KB represents knowledge as a network and we applied this concept to explicitly state our knowledge about a model, the model purpose, the model parameters visible to the outside (input/output parameters) as well as patient related parameters such as age or blood pressure. We structure knowledge into objects and their relations. These formal concepts enables the use of controlled vocabularies to unambigously describe the *meaning *and purpose of a parameter, creating a semantic description. Based on these it is possible to detect *similarity *between the described parameters and, after manual verification, store the connection explicitly. We can search for patient related data that is relevant as input parameter value of a computational model. We can validate simulation outputs against individual or aggregated clinical data.

The entire system is based in the *model-view-controller *(MVC) architecture for web applications [16] and in the following subsections we will describe the functionalities and structure of the main modules of the SE.

### The simulation workflow management system (SWoMS)

The SWoMS monitors the correct functioning of the system and it is composed of two modules: The *data warehouse manager *and the *control module*.

The *data warehouse manager *is responsible for executing all the requests to the *SE Data-base *or to the KB, ensuring the correct and consistent information flow among modules. This module requests and retrieves information from the KB using a web service. Information retrieved from the KB, or sent to the KB or models, is codified under the communication language specified in the interoperability section.

**Figure 2:**
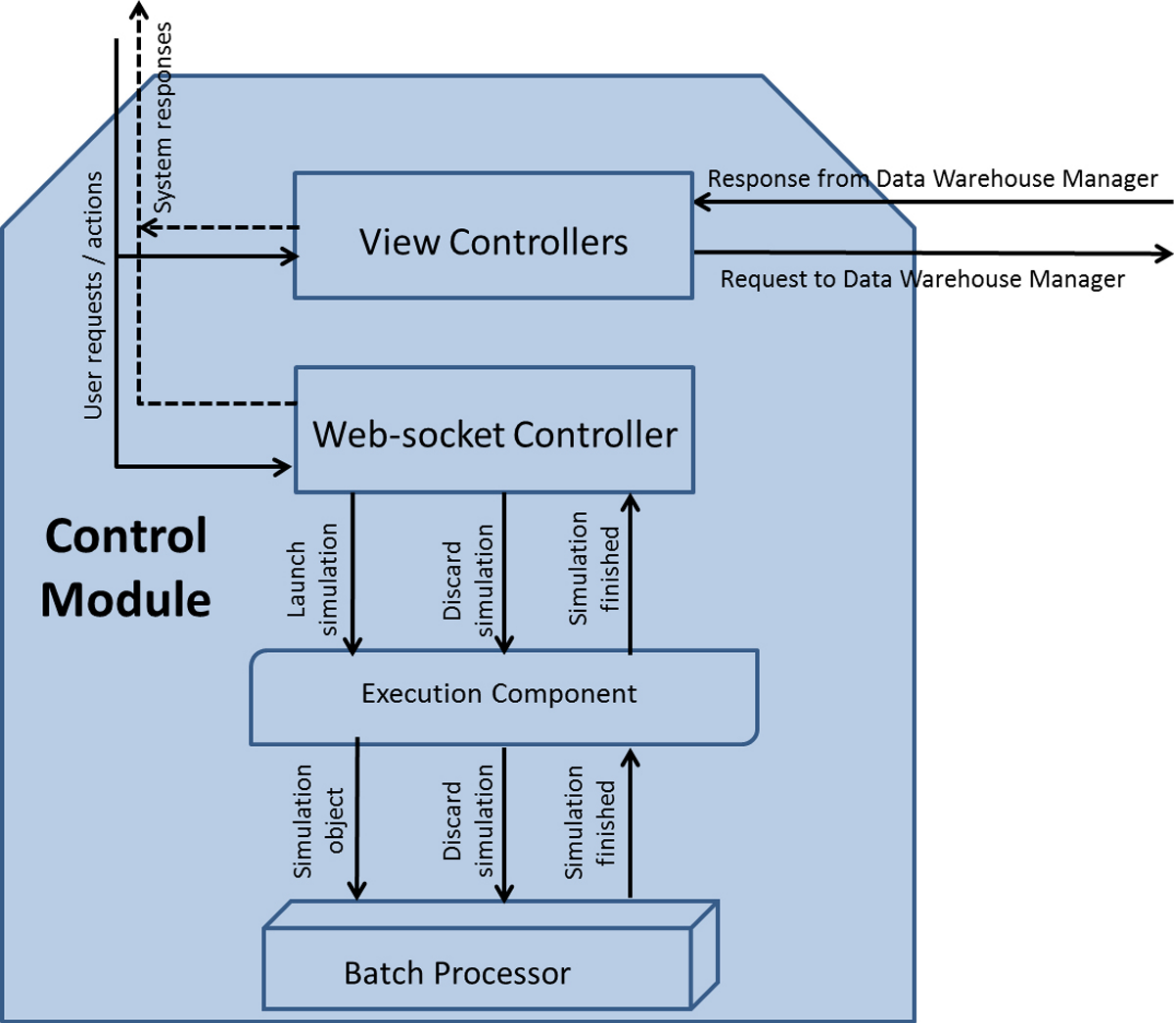
**Control module structure**.

The *control module*, presented in Figure 2, controls the correct functioning of the whole system. This module is composed of controllers. They receive user's actions, executed through the GVE, and respond to those actions. Controllers are divided in two groups: *view controllers *and *web-socket controller*. The *view controllers *receive those user's actions corresponding to requests to retrieve information from the *data warehouse manager*. This sends the result of those queries to be presented in the GVE. The *web-socket controller *receives user's actions to start or discard simulations. It also receives notifications from the *execution controller *to be sent to the GVE via a web-socket [17].

**Figure 3:**
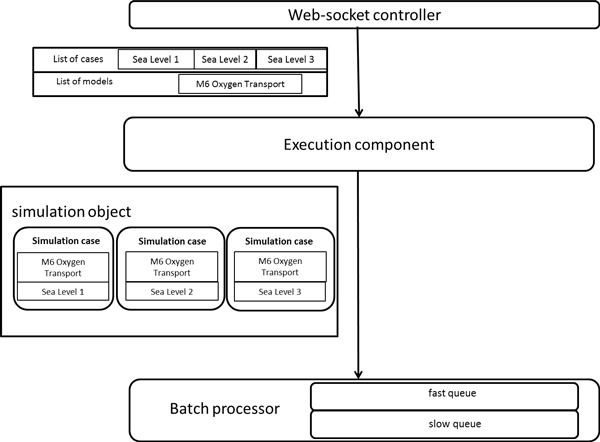
**Sequence followed by the list of cases and models to start a simulaiton from the web-socket controller to the batch processor**.

Among the information sent to the *web-socket controller *to start a simulation, we have one or more models to execute and one or more cases, both selected by the user. With the information received, the *execution component *creates a *simulation object*. The *simulation object *contains the logics to allocate the cases with the models to create a *case simulation*. A *case simulation *represents the minimum execution unit composed of a reference to the model to execute and the case, which contains the input parameters for the model. For example, in Figure 3 the user has selected a model called *M6 Oxygen Transport *and a list of cases which includes *Sea Level 1, Sea Level 2 *and *Sea Level 3*. These are sent to the *batch processor *which will forward them to the *execution component*. The *execution component *will create the *simulation object *with that model and that list of cases. The result of the execution of the *M6 Oxygen Transport *model with every case in the list of cases is displayed in the GVE.

Every *simulation object *has a complexity associated. This complexity is calculated considering the number of *case simulation *to execute by a model and the number of models to run. The complexity tends to be higher as the number of models and *case simulation *increase.

The *batch processor *is composed of two different queues: the *fast queue *contains *simulation objects *whose complexity is under a specific limit and the *slow queue *contains those *simulation objects *whose complexity is over a specific limit. For instance, the simulation object presented in Figure 3 will be directed to the *fast queue*.

When the execution of a *simulation object *has finished a finalization event is sent to the *web-socket controller *which will send the appropriate notification to the GVE.

### The graphical visualization environment (GVE)

**Figure 4:**
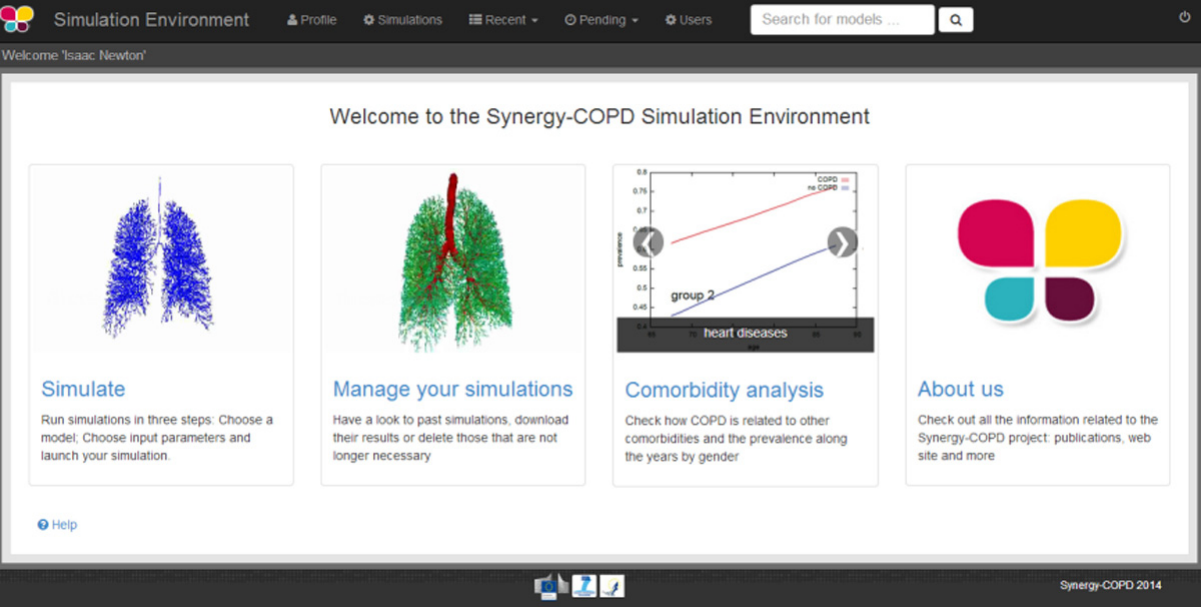
**Home of the simulation environment**.

The GVE, presented in Figure 4, is the front-end of the SE. Only those who are registered in the system can have access to the SE. The SE supports three different user profiles:

• *Administrator *is the only profile that has full access to all the possible actions that can be performed in the SE.

• *Modeller *is able to see pre-existing models, simulate them and map models if they have a mapping parameter. He will be able to upload and publish his own models following the standard described in Synergy-COPD to keep consistency with the different modules that are part of the Synergy-COPD project.

• *Doctor *is able to see pre-existing or public models included in the Synergy-COPD simulation environment, map models, if they have a mapping parameter, and run simulations modifying data in the cases.

Table 2 presents all the actions that every profile can perform in the SE.

**Table 2:** actions and permission related to each user profile, where 'Y' stands for Yes and 'N' stands for No.

Actions	Profile: administrator	Profile: doctor	Profile: modeller
**Create a user**	Y	N	N
**Assign a profile**	Y	N	N
**Modify user data**	Y	Y	Y
**View a model**	Y	Y	Y
**Run pre-existing and public models**	Y	Y	Y
**Run pre-existing and own models**	Y	N	Y
**Map models**	Y	Y	Y
**Make a model available to the public**	Y	N	Y

**Figure 5:**
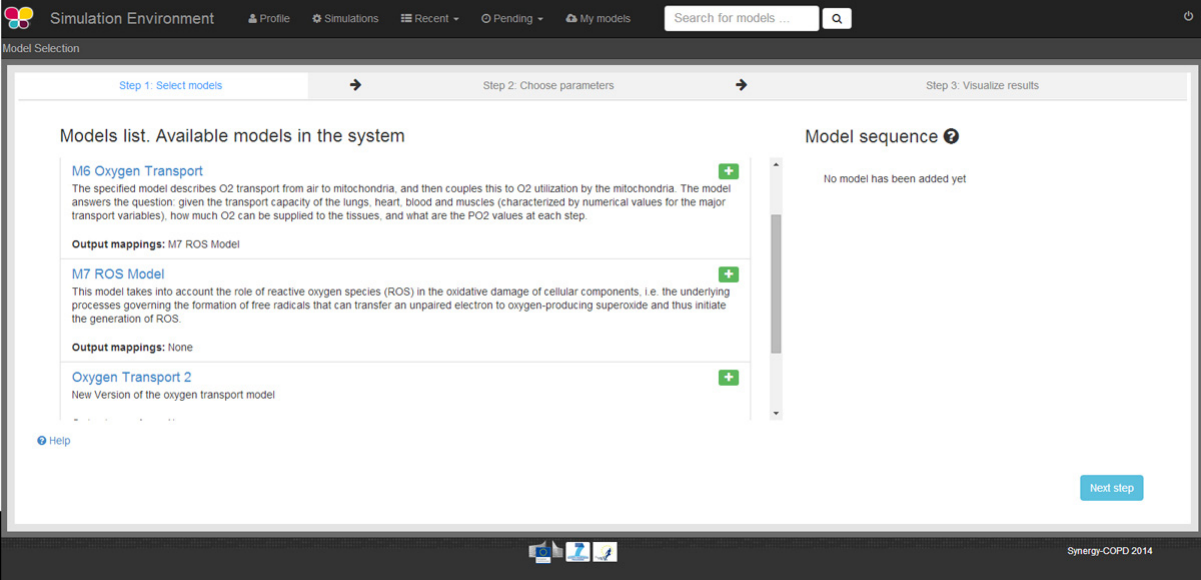
**Once the user has logged in, s/he can see the models available in the system or look for them introducing a keyword on the top right side of the screen**.

Once the user is logged in, s/he can see the models included in the system (see Figure 5), or search for models in the KB by introducing a keyword that is present in the models' metadata. When the user selects a model to be executed, another model can be added to the execution queue provided that a mapped parameter exists between them.

**Figure 6:**
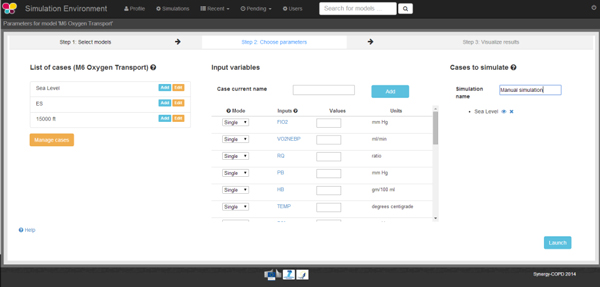
**On the left hand side of the image we can see the list of default cases associated to this model**. In the center there is a table with the list of variables included in the model and, on the right hand side, we can see the list of models that would be executed with the model.

The next step is to select one or more cases to be executed with that model or create a new one. If a case is edited, the values assigned to the variables of that case are shown. The user can: (1) change variables value or (2) introduce a range of values, within the *permitted range of values *(PRV) or (3) add the case into the queue of cases to be executed. Figure 6 shows that the user has selected *M6 Oxygen Transport *model and the cases labeled *Sea Level 1, Sea Level 2 *and *Sea Level 3*. Once the user has finished selecting cases to run, the launch button is used to send the information to the correspondent module as presented in Figure 3 and trigger the execution of the model.

**Figure 7:**
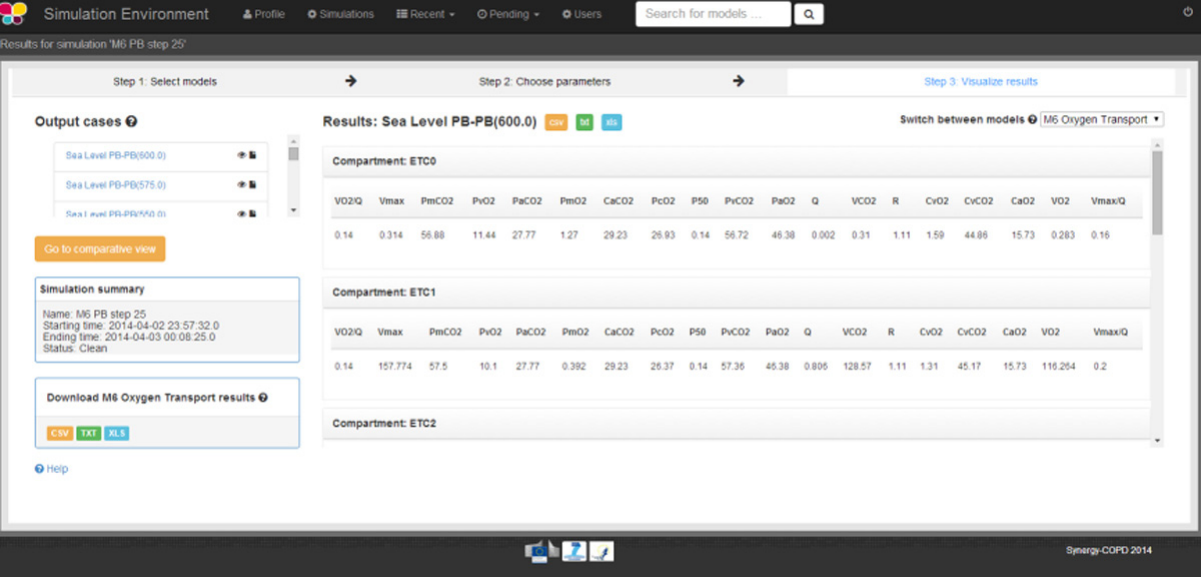
**On the left hand side of this image we can see the list of output cases generated after model executioni and, on the right hand side, we can see the output variables with the values after model execution for every output case**.

**Figure 8:**
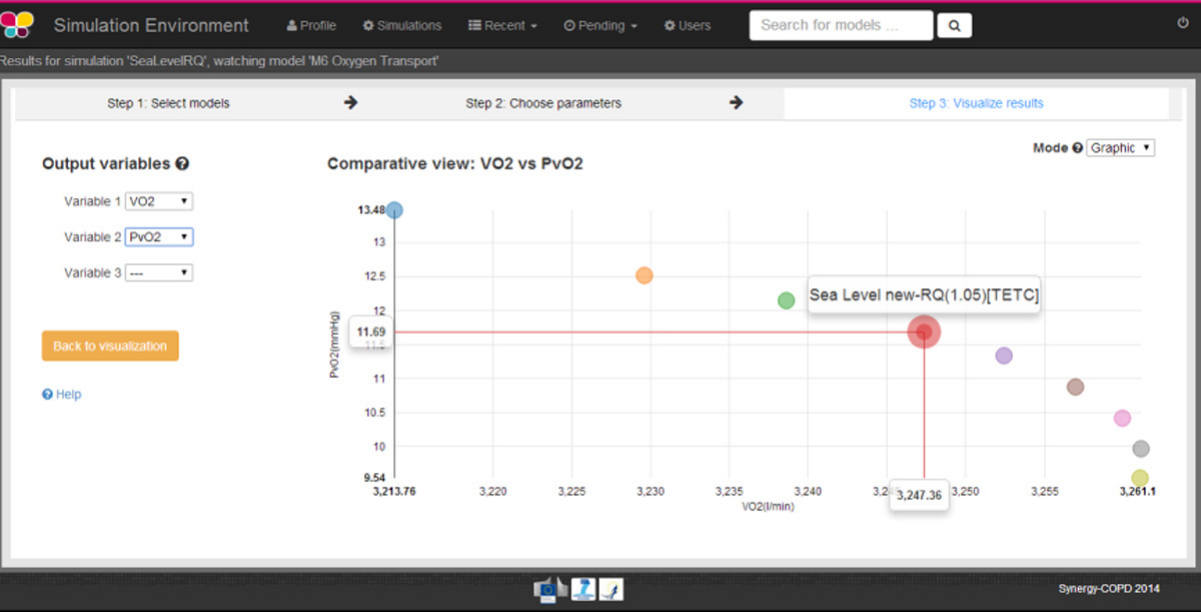
**On the left hand side of this image we can see that the user can choose up to three output variables of the model to be plotted**. Our user has selected VO2 and PvO2. On the right hand side, we can see the graphical representation of these two output variables.

A visual alert is presented when a simulation has finished. The results after model execution may be presented in tabular format (see Figure 7) or as a graphical plot (see Figure 8). The results may be downloaded in various formats including csv, spreadsheet and plain text format. The GVE also supports case storage and case management.

For a live demo please visit the youtube channel of the Synergy-COPD Simulation Environment (https://www.youtube.com/watch?v=dZQO8qRYMac).

## Interoperability

Interoperability in the SE is defined as the capability of exchanging parameters between the SE, the KB and the models in a consistent manner. This communication needs to be done in a simplified way to allow model creators to integrate the models descriptions in the KB facilitating the communication of parameter values. To formalize such communication, a communication language denominated SE-TAB was defined.

This communication language has been developed after reviewing existing model description languages and standards for minimum information reporting. Following the success of simple tab-delimited formats for gene expression [18] and other *omics *data [19] we generated SE-TAB which allows model creators to generate very simple wrappers for their model parameter input/output values. To add models description and model parameters to the KB we can use the SE-TAB language or other formats. Therefore the absolute minimum SE-TAB support for integration into the SE is the parameter value file which any model needs to be able to read and produce.

As models are uniquely identified within the SE, the communication can be based on model parameter names. Parameters, for which different values occur in different spatio-temporal compartments, are listed only once, the values being reported as array indexed by the corresponding spatio-temporal compartment information (which is specified in the corresponding model and model parameter description), thus enabling an extremely simple format as shown in Table 3.

**Table 3:** Example of SE-TAB format.

Parameter name	Value (;delimited array)	Spatio temporal index
**vcitSyn**	2.31;2.40	l1:t1;l2:t1
**cmal_o**	0.53;0.65	l1:t1;l2:t1

**Table 4:** Table of definitions.

Concept	Description
**Interoperability **	Capability of exchanging parameters between models in the SE

**Ontology **	It is a way of storing information based on the relations of the different entities included in an ontology

**Mapping parameters **	Those parameters that have different representation in different models, although they are the same.

**Semantic description **	Description of a concept by meaning

**Web service **	It is a communication method to exchange data or information between applications

**Web socket**	Makes possible the communication between a client application (web browser) and a server (remote machine). In this type of communication a channel is open to send and receive data from both parts.

**wrapper **	Piece of code that works as intermediary between two source codes written in different programming languages.

## Workflow

**Figure 9:**
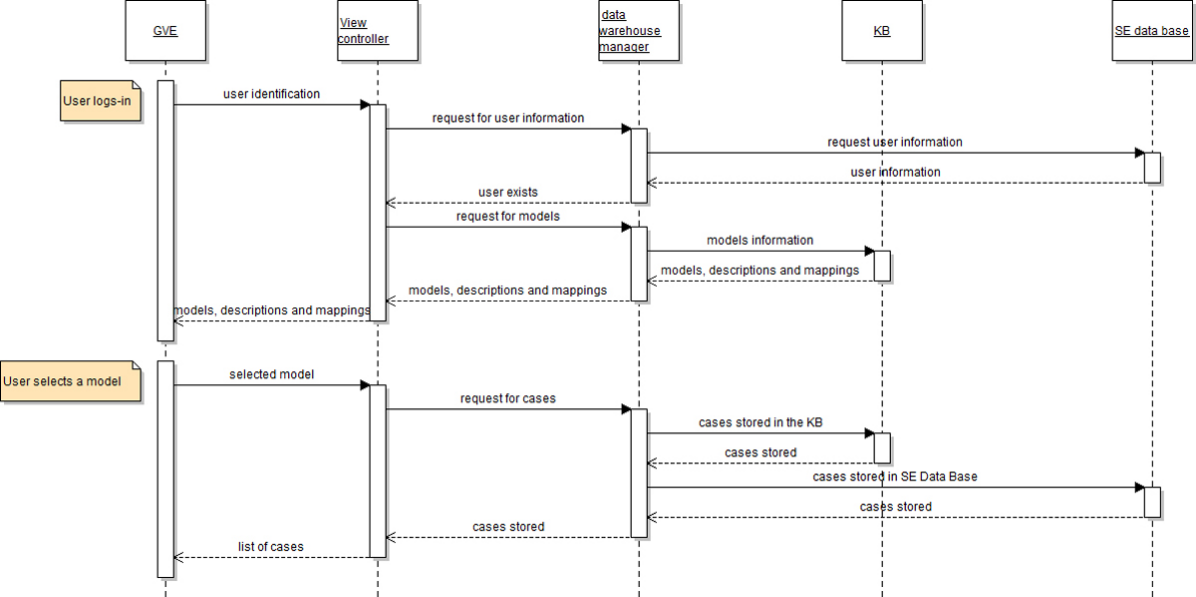
**View controller sequence diagram**.

A typical workflow in the SE is shown in Figure 9. First the user logins and credentials are verified. Next the *view controller *pulls from the KB all the models, associated description, the semantic meta-information and the models mapped parameters. This information is processed in the *view controller *and presented to the user through the GVE. When the user selects a model, the *view controller *via the *data warehouse manager *pulls all the associated cases from the KB and the *SE Data-base*. This information is eventually presented to the user by GVE.

**Figure 10:**
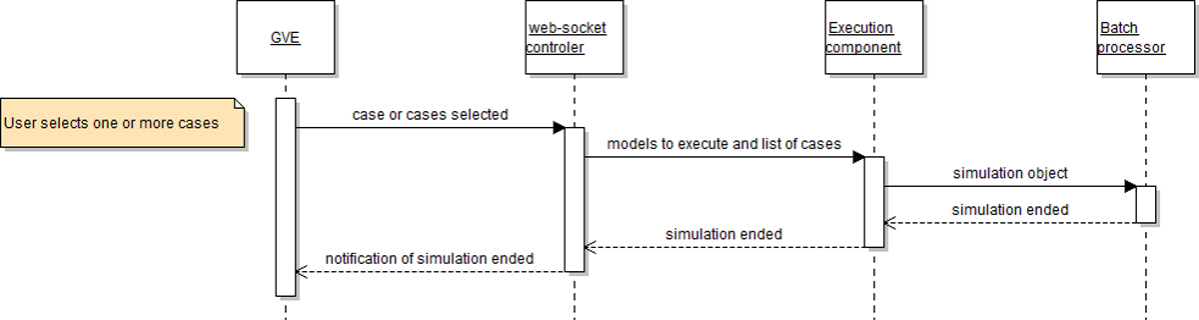
**Web-socket simulation launch sequence diagram**.

Once the user has selected one or more cases, as shown in Figure 10, the GVE will send the information, via the web-socket *controller*, arriving at the execution *component *to create a simulation *object*. This simulation *object *will be inserted in one of the two queues of the batch *processor*. When the simulation finishes, the user will be notified.

## Validation

The SE was validated by utilizing COPD-relevant physiological models. The capabilities to execute models in different programming languages, to store cases and the communication capabilities with the previously described communication standard were extensively tested.

The models utilized belong to two different paradigms: deterministic and probabilistic. The first of the two deterministic mathematical models considered in the Synergy-COPD project have been recently extended from a prior model describing O2 transport from air to mitochondria as an integrated system limiting maximal oxygen uptake $V_{O_{2}MAX}$[6,20] to also include the contribution to overall impedance to O2 flow from the above-zero mitochondrial oxygenation levels $\left(P_{mo_{2}}\right)$ required to drive mitochondrial respiration [7]. This has enabled the prediction of $P_{mo_{2}}$ as a balance between the capacities for muscle O2 transport and utilization [7]. This model has been recently expanded to allow functional heterogeneity in both lungs and muscle. This was done to enable application to disease states. This model, called *M6 Oxygen Transport*, is currently programmed using Java programming language.

The second deterministic model considered in Synergy-COPD provides a conceptual basis for the abnormally high reactive oxygen species (ROS) production observed both in hyperoxia [21,22] and hypoxia [23-26] and has the ability to predict the quantitative relationship between ROS generation and $P_{mo_{2}}$. Recent modeling and experimental studies on mitochondrial ROS production under hypoxia and re-oxygenation [8,27,28] have proposed an inherent bi-stability of Complex III, i.e. coexistence of two different steady states at the same external conditions: one state corresponding to low ROS production, and a second potentially dangerous state with high ROS production. Temporary deprivation of oxygen could switch the system from low to high ROS production, thus explaining the damaging effects of hypoxia-re-oxygenation. This model, called *M7 Cell bioenergetics, mitochondrial respiration and ROS generation*, was developed in the C++ programming language.

With the aim to offer the researcher a more COPD and patient specific approach, a *Bayesian network *was developed [1] to approach parameter specification. The creation of a *Bayesian network *requires a lot of data in order to validate it. Due to the lack of data to validate this network, we have proposed a *Bayesian network *for those researchers who want to apply expert knowledge or new data. The *Bayesian network *was developed in the C++ programming language.

**Figure 11:**
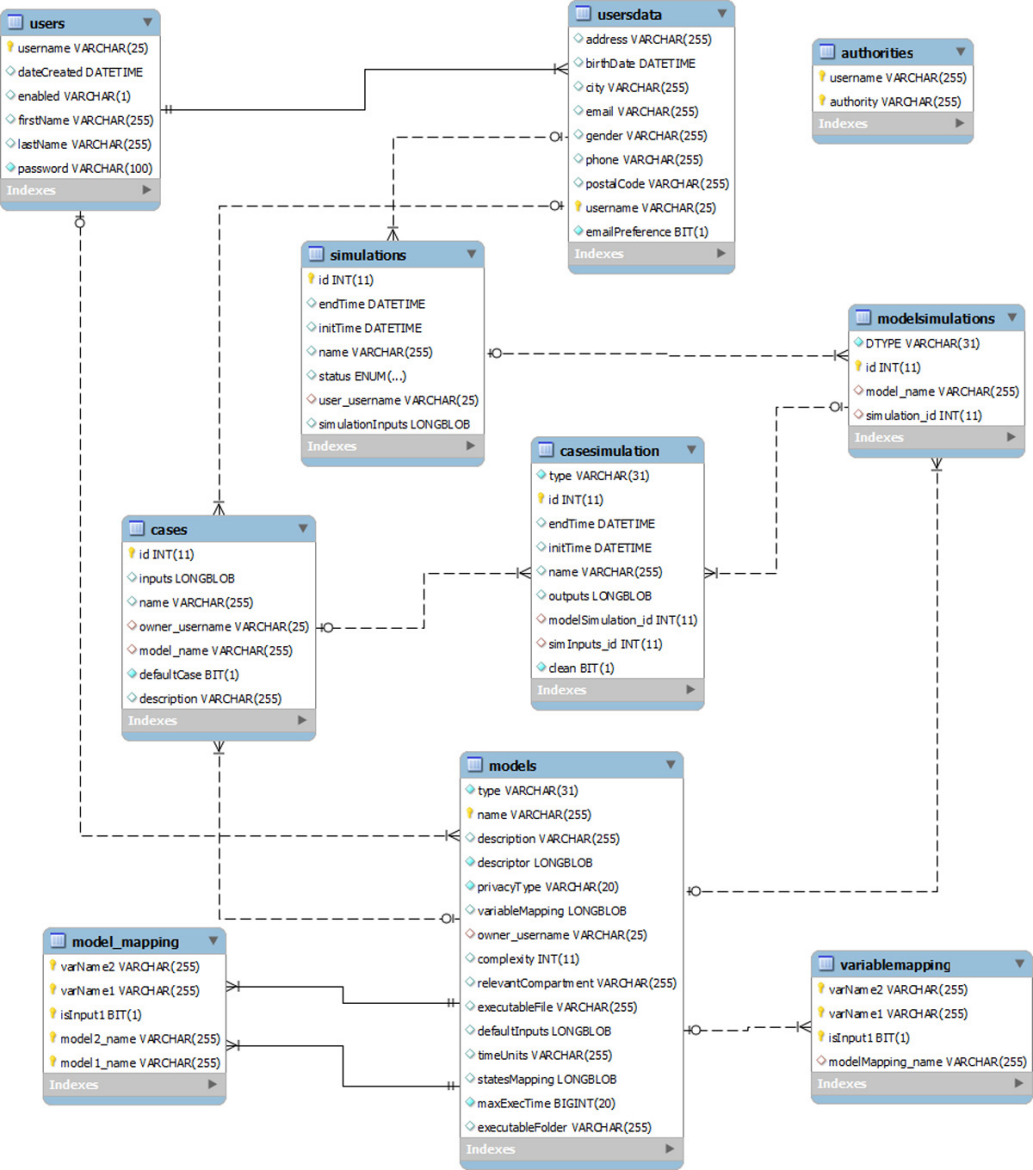
**SE data base schema**.

Cases, such as *Sea Level *and *SE*, visible on the left hand side of Figure 6, are stored in the KB. New cases can be created using the two as a starting point, and can be stored in the *SE Data-base *whose schema is presented in Figure 11.

## Conclusions and future work

In this work we have presented a web-based simulation environment composed of two main modules: a user interface and a simulation engine responsible for controlling the correct functioning and flow of information among the different actors included in the system. These actors are: the user, the KB and the physiology models. The SE was validated with physiology models related to COPD.

In the current status of the SE the integration among static models was achieved although it does not allow running models with different temporal dimensions therefore we plan to extend the SE to support this feature.

An objective that was originally planned was the personalization of the results thanks to the use of the probabilistic model that could use patient specific data. However, this personalization was not achieved due to the lack of evidences that could allow the connection among the probabilistic model and the deterministic models in a consistent manner.

A feature added recently to the SE is the possibility to upload new models in the system turning the SE into a collaborative platform. A user with modeler permissions can upload a model and test it. By making it public other modelers registered in the system could use it and build other models if there are existing mappings between models. Making the SE available to the general public will allow users to share results and models.

The specialization of the tool to be employed as an educational tool is also under consideration so that the SE can be utilized by medicine students who have a clear interest in research about the respiratory chain.

A co-operation is planned with related projects that may benefit from this SE. A specific case is the AirPROM FP7 project [29] which is developing VPH models for lungs at cell and organ levels. Those models might be a great extension of those developed in the Synergy-COPD project and a co-operation between both projects has been initiated. This is an opportunity to allow the SE to continue its evolution in the framework of AirPROM.

The source code for the SE is available under Lesser General Public License in a public repository. [30]

## Competing interests

MMHM, DMo, MLA and FM are employed at Barcelona Digital Centre Technologic and will therefore be affected by any effect of this publication. IC, DMa and DGC work in different academic institutions and SMEs and will therefore be affected by any effect of this publication on their publication records. DM is employed at Biomax Informatics AG and will therefore be affected by any effect of this publication on the commercial version of the BioXM™ Knowledge Management Environment on which the mentioned COPDKB is based.

## Authors' contributions

MMHM conceived and designed the initial SE. MMHM was the lead developer and research engineer of the SE. DMo worked in the generalization phase of the SE. MLA contributed to the design, improvements and refinements in the last stages of the SE. IC and DGC provided expertise in modelling, model integration and physiology. DMa, IC and MMHM co-developed the communication language for parameter exchange. DMa supported the integration of the KB in the SE architecture. FM as coordinator of the Synergy-COPD project and reviewer of the manuscript. All authors read and approved the final manuscript.

## Declaration

The research described in this manuscript is supported by the Synergy-COPD European project (FP7-ICT-270086). The opinions expressed in this manuscript are those of the authors and are not necessarily those of Synergy-COPD project's partners or European Commission.

This article has been published as part of *Journal of Translational Medicine *Volume 12 Supplement 2, 2014: Systems medicine in chronic diseases: COPD as a use case. The full contents of the supplement are available online at http://www.translational-medicine.com/supplements/12/S2.
